# Experimental Model of Pulmonary Inflammation Induced by SARS-CoV-2 Spike Protein and Endotoxin

**DOI:** 10.1021/acsptsci.1c00219

**Published:** 2022-01-25

**Authors:** Manoj Puthia, Lloyd Tanner, Ganna Petruk, Artur Schmidtchen

**Affiliations:** † Division of Dermatology and Venereology, Department of Clinical Sciences, Lund University, SE-22184 Lund, Sweden; ‡ Division of Respiratory Medicine and Allergology, Department of Clinical Sciences, Lund University, SE-22184 Lund, Sweden; § Bispebjerg Hospital, Department of Biomedical Sciences, University of Copenhagen, DK-2400 Copenhagen, Denmark

**Keywords:** COVID-19 *in vivo* models, SARS-CoV-2 spike (S) protein, ARDS, LPS, TCP-25

## Abstract

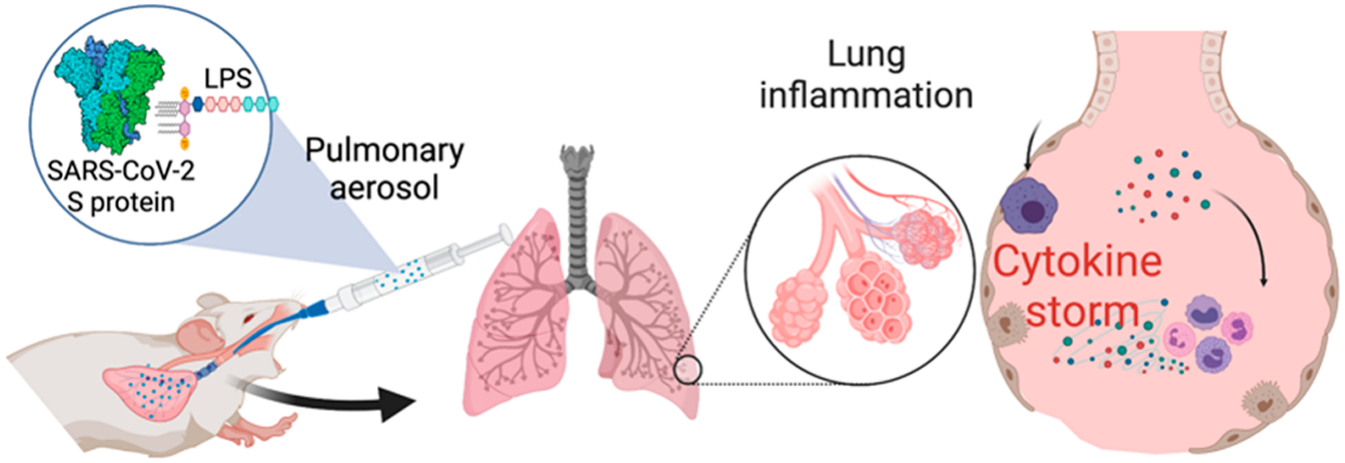

COVID-19 is characterized by a dysregulated and excessive inflammatory response and, in severe cases, acute respiratory distress syndrome. We have recently demonstrated a previously unknown high-affinity interaction between the SARS-CoV-2 spike (S) protein and bacterial lipopolysaccharide (LPS), leading to the boosting of inflammation. Here we present a mouse inflammation model employing the coadministration of aerosolized S protein together with LPS to the lungs. Using NF-κB-RE-Luc reporter and C57BL/6 mice followed by combinations of bioimaging, cytokine, chemokine, fluorescence-activated cell sorting, and histochemistry analyses, we show that the model yields severe pulmonary inflammation and a cytokine profile similar to that observed in COVID-19. Therefore, the model offers utility for analyses of the pathophysiological features of COVID-19 and the development of new treatments.

Infection with SARS-CoV-2 yields a spectrum of symptoms, from fever, cough, dyspnea, and myalgia to severe disease such as acute respiratory distress syndrome (ARDS) and septic shock. Animal models in mice, hamsters, ferrets, and nonhuman primates have been developed to study mechanisms and therapies.^1−4^ Because mouse ACE2 does not effectively bind the viral spike protein, transgenic mouse models employing the expression of human ACE2 are used.^5,6^ These mice are susceptible to infection by SARS-CoV-2, albeit with differences in disease severity. It is of note that at present, no mouse model recapitulates all aspects of COVID-19 in humans, especially pulmonary vascular disease and ARDS. Because of the multifaceted nature of the disease, there is a need for new animal models that enable us to study both general and specific aspects of COVID-19 pathogenesis and treatment.

During ARDS, the activation of toll-like receptors (TLRs) such as TLR4 via lipopolysaccharide (LPS) stimulation induces an initial systemic pro-inflammatory phase characterized by a massive release of pro-inflammatory mediators such as cytokines/chemokines and the activation of proteolytic cascades, including the coagulation and complement system.^7,8^ Therefore, the clinical symptoms of patients with ARDS in many ways correspond to the pathophysiology seen during severe COVID-19 disease. Certain comorbidities characterized by increased LPS levels, such as diabetes, obesity, and chronic obstructive pulmonary disease (COPD), predispose patients to ARDS during SARS-CoV-2 infection.^9−12^ We have recently demonstrated a previously unknown high-affinity interaction between the SARS-CoV-2 S protein (here denoted as S protein) and LPS from *E. coli* and *P. aeruginosa*, leading to a hyperinflammation *in vitro* as well as *in vivo*. The molecular mechanism underlying this effect was shown to be dependent on specific and distinct interactions between the S protein and LPS, enabling LPS’s transfer to CD14 and subsequent downstream NF-κB activation.^12^ The resulting synergism between the S protein and LPS has clinical relevance, providing new insights into comorbidities that may increase the risk for ARDS during COVID-19. An experimental model that recapitulates this particular facet of COVID-19 could therefore aid in addressing ARDS pathogenesis and provide a platform for evaluating new therapeutics. To this end, we present a mouse model employing the coadministration of the aerosolized SARS-CoV-2 S protein and bacterial LPS to the lungs, producing severe pulmonary inflammation and a cytokine profile sharing features observed during COVID-19 disease progression.

To investigate whether the S protein can activate TLR-mediated inflammation in the lungs, we used NF-κB-RE-Luc reporter mice. The S protein was administered intratracheally to murine lungs using a specialized pulmonary aerosol delivery device (Figure 1A). Exposure to 5 μg of S protein alone did not induce significant NF-κB expression, with relatively low levels of bioluminescence emission recorded from the pulmonary regions 6 and 24 h after administration of the protein (Figure 1B). As expected, 2 μg of LPS alone showed a similarly low NF-κB induction. Interestingly, when a combination of 5 μg of S protein and 2 μg of LPS was delivered to the lungs of the mice, we observed a significantly increased NF-κB induction (Figure 1B). Because a part of the bioluminescent signal appeared to originate from outside the thoracic cavity, we performed *ex vivo* IVIS imaging of the lungs, liver, and kidneys (Figure S1A,B). The results showed that a major part of the bioluminescence signal detected in mice administered with S-protein+LPS originated from the lungs. A low level of NF-κB induction was also observed in the liver and kidneys. To further substantiate if the enhanced NF-κB induction was due to the S protein and LPS synergism, we added TCP-25, a thrombin derived peptide that acts as an LPS scavenger and CD14 blocker.^13^ Initial *in vitro* data demonstrated that TCP-25 bound to LPS in a dose-dependent manner (Figure S2A) and abolished LPS binding to the S protein because the intensities of the bands at ∼480 kDa for the S-protein+LPS+TCP-25 and the S protein alone were similar (Figure S2B). Functionally, TCP-25 abrogated S-protein-mediated boosting of LPS responses (Figure S2C). *In vivo*, TCP-25 abolished the S-protein+LPS-induced activation of NF-κB in murine lungs, demonstrating that the observed effects on inflammation are LPS-dependent (Figure 1B).

**Figure 1:**
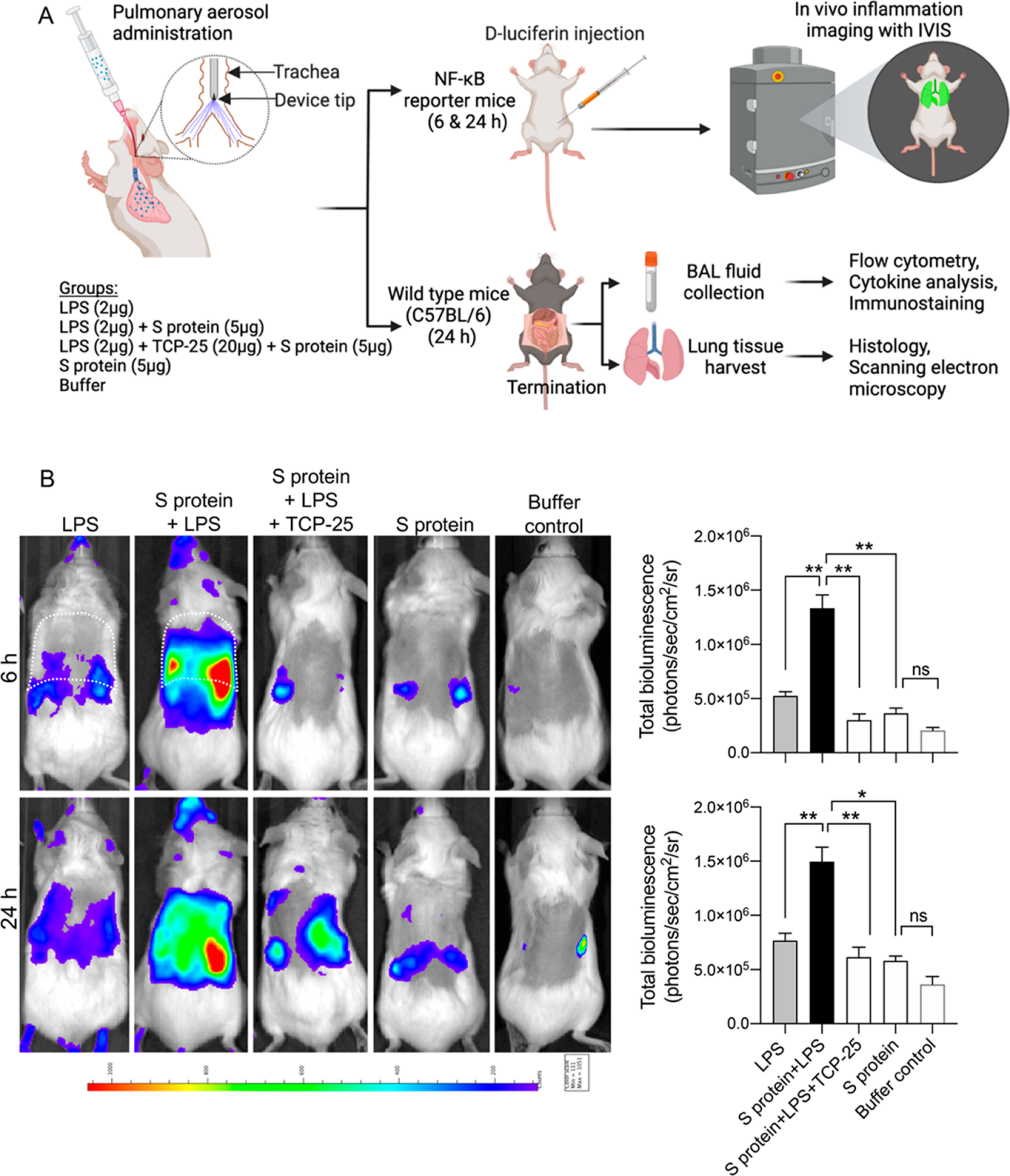
*In vivo* imaging of pulmonary inflammation in NF-κB reporter mice. (A) *In vivo* experimental plan in mice. LPS alone, in combination with the SARS-CoV-2 S protein (S protein), was intratracheally administered in transgenic BALB/c Tg(NF-κB-RE-luc)-Xen reporter mice. TCP-25, a thrombin-derived peptide, was also used as an LPS scavenger. BioRender was used for illustrations. (B) Longitudinal noninvasive *in vivo* bioimaging of NF-κB reporter gene expression was performed using IVIS imaging. Representative images show bioluminescence at 6 and 24 h after intratracheal administration. The bar chart shows the bioluminescence intensity acquired from these reporter mice. The white dotted line indicates the borders of thoracic cavity. Data are presented as the mean ± SEM (*n* = 4 mice for the LPS group, 4 mice for the S-protein+LPS group, 4 mice for the S-protein+LPS+TCP-25 group, and 3 mice for the S-protein group). *P* values were determined using a one-way ANOVA with Tukey’s posttest. *P ≤ 0.05, **P ≤ 0.01. ns, nonsignificant.

We further investigated if S protein and LPS combination-induced pulmonary NF-κB expression corresponds to inflammatory cell infiltration in the lungs. Aerosolized S-protein+LPS was delivered intratracheally in C57BL/6 mice, and the bronchoalveolar lavage fluid (BALF) was analyzed using flow cytometry. Given the propensity for LPS to facilitate immune cell recruitment,^14^ flow cytometry was conducted on murine BALF samples to allow for the quantitative determination of neutrophils, alveolar macrophages, and inflammatory macrophages, with representative gating shown in Figure 2A. Following the administration of intratracheal LPS, an increase in murine BALF neutrophils was recorded compared with the S protein and vehicle control samples (Figure 2B). Interestingly, a significant increase in both inflammatory macrophages and neutrophils was recorded in S-protein+LPS-exposed lungs when compared with LPS alone. Fluorescence microscopy conducted on murine BALF further supports these findings, with increased CD206 staining in the samples from lungs subjected to the S protein and LPS combination (Figure 2C) indicative of M2/activated macrophages.^15^ Enlarged inflammatory macrophage nuclei were observed in the smear preparations of BALF material derived from S-protein+LPS-treated animals. This finding adds to the work conducted by Petruk et al. and further elucidates the inflammatory response induced by the lung-specific interaction between LPS and the SARS-CoV-2 S protein.^12^

**Figure 2:**
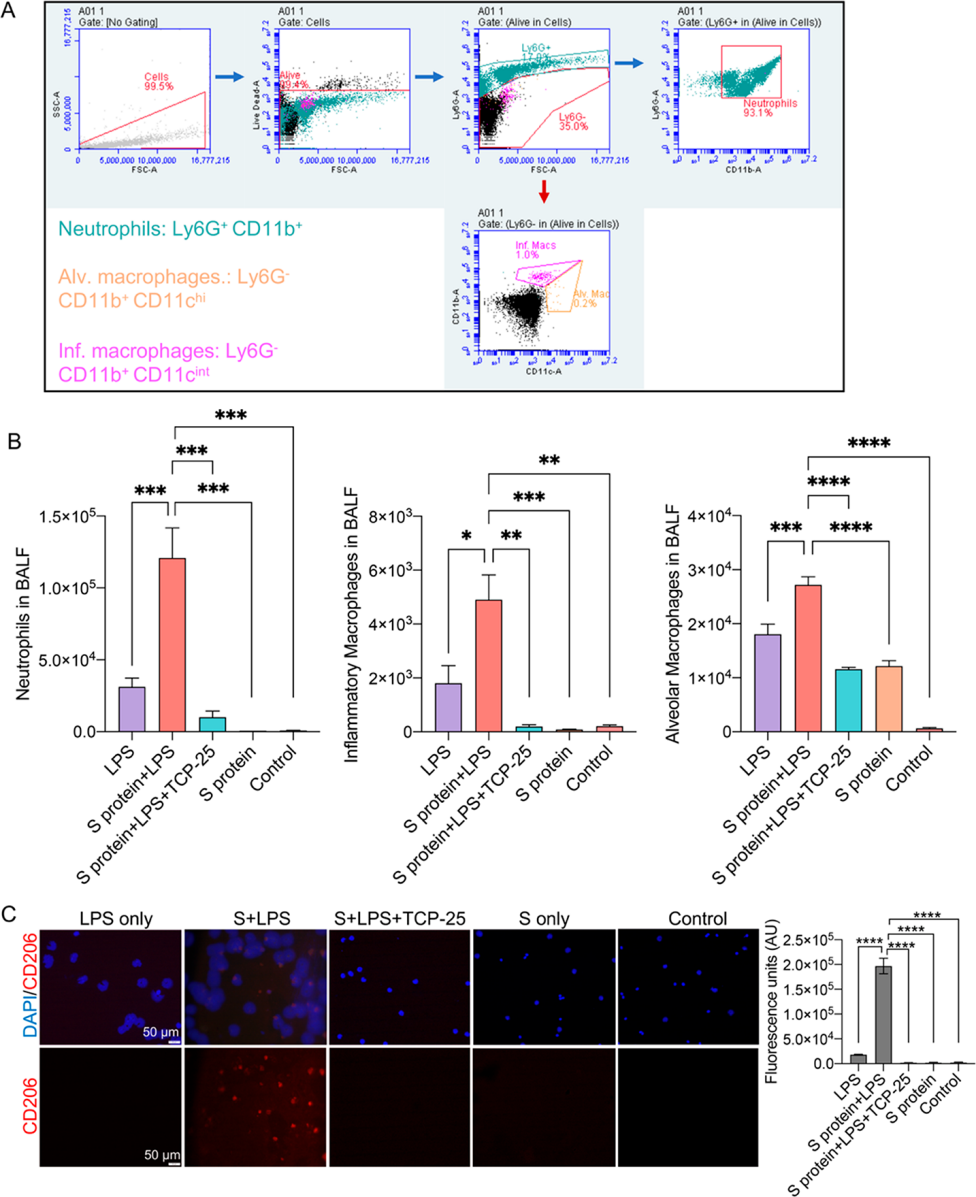
Flow cytometry analysis and immunostaining of bronchoalveolar lavage fluid (BALF) showing cellular infiltration in the lungs. (A) Representative gating strategy for the flow cytometry analysis of BALF. (B) Bar charts showing numbers of neutrophils, inflammatory macrophages, and alveolar macrophages in BALF. Data are presented as the mean ± SEM (*n* = 4 mice for the LPS group, 5 mice for the LPS and S-protein group, 3 mice for the S-protein+LPS+TCP-25 group, 3 mice for the S-protein group, and 3 mice for the buffer control). *P* values were determined using a one-way ANOVA with Dunnett’s posttest. (C) CD206 staining of cytospin smears of BALF showing inflammatory macrophages (red). DAPI was used as a nuclear counterstain (blue). Representative fluorescence microscopy images are shown. The bar chart shows the CD206 fluorescence intensity measured from the stained smears. Data are presented as the mean ± SEM (*n* = 4 mice for the LPS group, 5 mice for the S-protein+LPS group, 3 mice for the S-protein+LPS+TCP-25 group, 3 mice for the S-protein group, and 3 mice for the buffer control). *P* values were determined using a one-way ANOVA with Dunnett’s posttest. *P ≤ 0.05, **P ≤ 0.01, ***P ≤ 0.001, ****P ≤ 0.0001.

The presence of high numbers of inflammatory cells in BALF prompted us to investigate the cytokine patterns induced by the S protein and LPS combination. The analysis of BALF using a multiplex assay showed significant increases in the levels of several inflammatory cytokines and chemokines resembling a “cytokine storm” (Figure 3A,B). The concentrations of many of these inflammatory mediators were observed to be significantly decreased by TCP-25. The delivery of LPS or S protein alone to the lungs did not induce a cytokine storm, and the concentrations of inflammatory mediators in BALF were comparable to those found in the control group. Increases in IL-6, granulocyte-macrophage colony-stimulating factor (GM-CSF), tumour necrosis factor (TNF), and IL-18 have been observed in small BALF-based studies, in particular, in patients with severe COVID-19.^16,17^ A closer inspection of the multiplex results revealed increases in IL-6, GM-CSF, IL-1β, MIP-1α, MIP-1β, and other cytokines following S-protein+LPS administration to the lungs. Increased levels of IL-6 have been contested as drivers of COVID-19 disease progression following several failed IL-6 inhibitor trials.^18^ However, in combination with IL-1β, the IL-1/IL-6 axis plays a key role in immune cell infiltration,^19^ as supported by this model. Additionally, increased levels of MCP-1 and MIP-1α were present in patients with COVID-19 admitted to the emergency unit.^20^ Both MIP-1α and MCP-1 were increased in the S-protein+LPS-treated murine lungs, promoting the recruitment of alveolar and inflammatory macrophages. Moreover, the levels of other inflammatory mediators such as IL-1α, RANTES (regulated upon activation, normal T cell expressed and presumably secreted), IL-12 p40, and IL-12 p70 were also increased with the coadministration of the S protein and LPS (Figure S3). Taken together, the combined analyses of cytokines, chemokines, and cell populations in this experimental model demonstrated both a cytokine storm induction and immune cell infiltration, key features of severe COVID-19,^21−23^ adding further relevance to this murine model.

**Figure 3:**
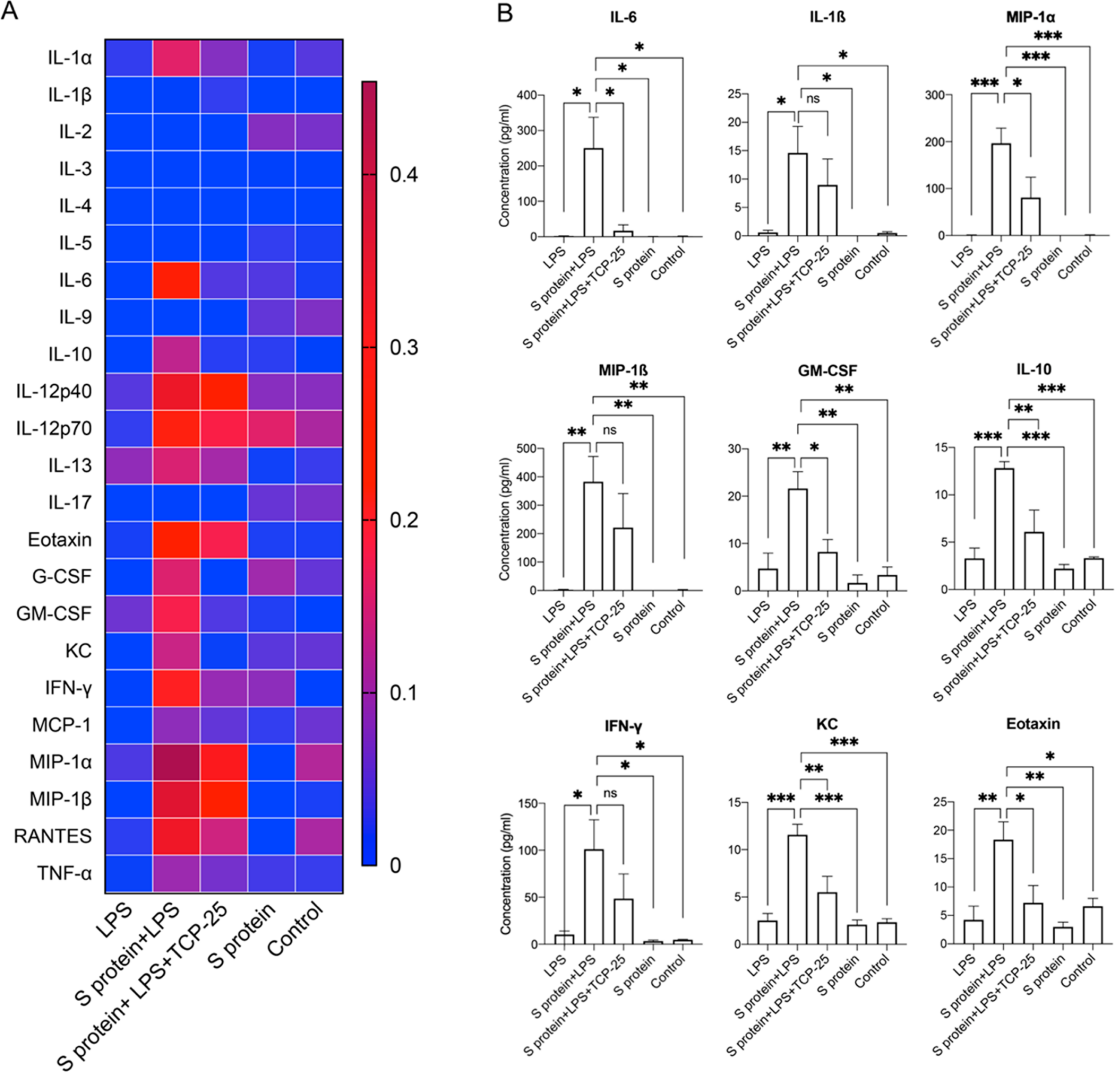
Bronchoalveolar lavage fluid (BALF) cytokine and chemokine patterns induced by the SARS-CoV-2 S protein and endotoxin. (A) Heat map showing changes in the cytokine and chemokine levels in BALF at 24 h, analyzed by a BioPlex Pro mouse cytokine assay. Mean normalized values for each cytokine readout are plotted in the heatmap (*n* = 3 mice for the LPS group, 4 mice for the S-protein+LPS group, 3 mice for the S-protein+LPS+TCP-25 group, 3 mice for the S-protein group, and 3 mice for the buffer control). (B) Bar charts showing inflammatory cytokines and chemokines significantly induced by the SARS-CoV-2 S protein and LPS combination. Data are presented as the mean ± SEM (*n* = 3 mice for the LPS group, 4 mice for the S-protein+LPS group, 3 mice for the S-protein+LPS+TCP-25 group, 3 mice for the S-protein group, and 3 mice for the buffer control). *P* values were determined using a one-way ANOVA with Dunnett’s posttest. *P ≤ 0.05, **P ≤ 0.01, ***P ≤ 0.001; ns, not significant.

Lastly, it was relevant to know if the cellular infiltration and cytokine storm caused by the S protein and LPS combination led to pathologic lung changes. Histological analysis of lung tissue sections showed severe hyperinflammation and degraded tissue architecture after exposure to the S protein and LPS combination (Figure 4A). Histological analysis yielded a significantly higher lung injury score in the group of mice that received the S protein and LPS. These pathologic tissue changes were confirmed with scanning electron microscopy of lung tissue (Figure 4B). As expected, significant numbers of alveolar macrophages were observed in the lung tissues from mice subjected to the S protein and LPS combination. These histological changes show similarities with the pathological lung changes in patients with severe COVID-19, where high numbers of neutrophils and macrophages are observed in the airways.^24^ Compatible with its inhibitory effects on LPS- and CD14-mediated signaling,^13^ TCP-25 reduced these pathological lung changes (Figure 4A,B). Recently, a TCP-25-functionalized hydrogel, reducing both excessive inflammation and bacteria, has been shown to be a promising therapy that promotes wound healing in preclinical wound infection models.^25,26^ Interestingly, the proof-of-principle data presented here suggest that the peptide could also be used to target hyperinflammation during COVID-19. TCP-25 was used here to demonstrate the LPS dependence of S-protein-mediated inflammation boosting. It should be noted, however, that separate TCP-25 interactions with the S protein cannot be excluded, and indeed, the occurrence of high-molecular-weight TCP-25 in the sample containing a mixture of TCP-25 and the S protein (Figure S2B) is compatible with such a binding. Therefore, in a reductionist approach and to minimize other possible confounding interactions, we decided to preincubate the peptide with LPS before mixing it with the S protein and administrating the mixture to the mice. Future studies should therefore address potential interactions between TCP-25 and the S protein in the presence of LPS and involve more complex animal models using TCP-25 administration. Although such studies are of clear therapeutic importance, they are outside the scope of the present work. In this context, it should also be noted that the present model mimics an acute situation, and future work should therefore address later-onset effects of the coadministration of the S protein and LPS and the effects of TCP-25. Moreover, because this work was mainly focused on pulmonary inflammation, future studies should also address aspects of inflammation propagation to other organs and the induction of systemic disease.

**Figure 4:**
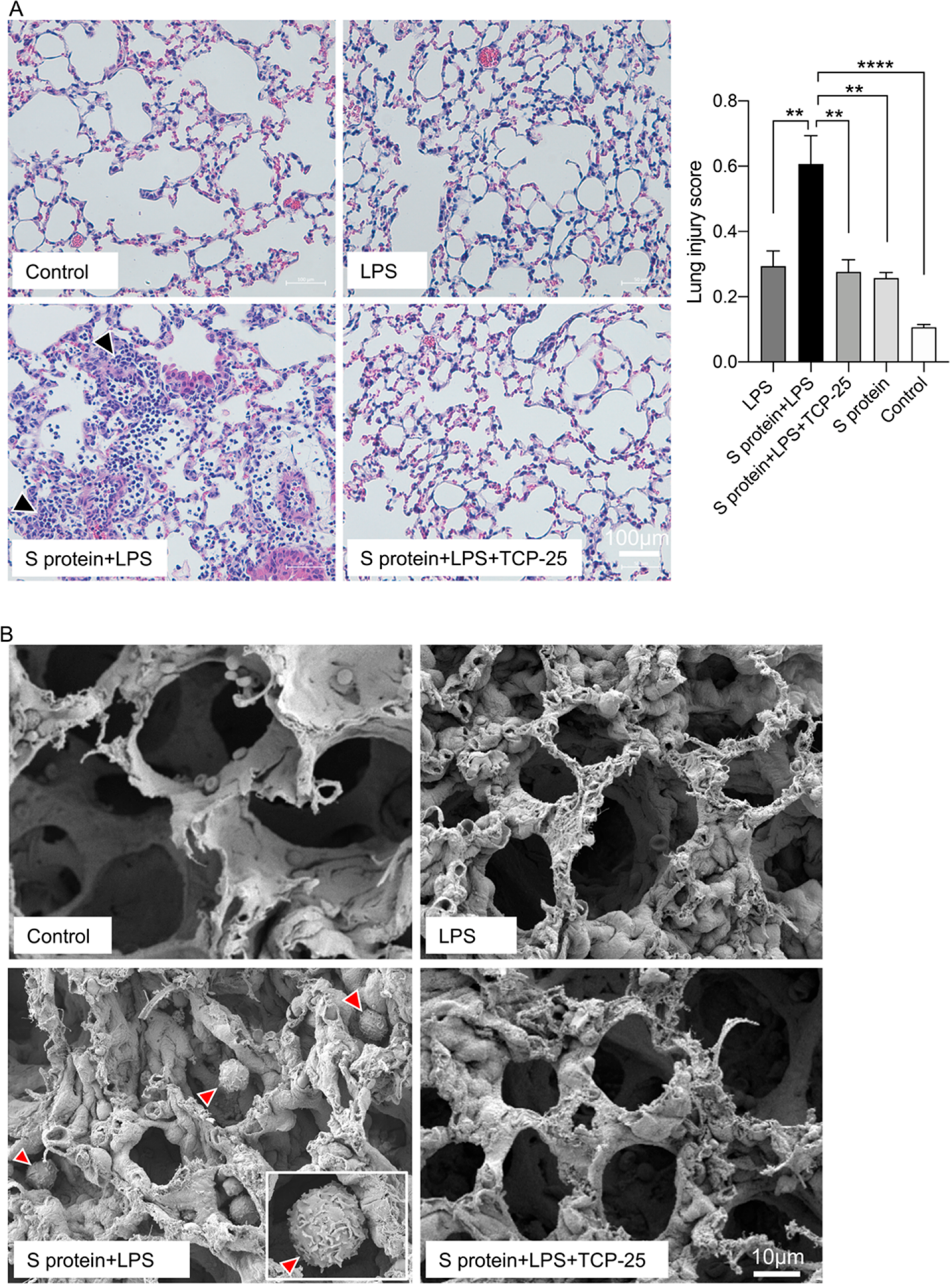
SARS-CoV-2 S protein and endotoxin-induced lung tissue changes. (A) Representative images showing H&E staining of mouse lung tissue 24 h after the intratracheal administration of the combination of SARS-CoV-2 S protein and LPS. Arrowheads indicate areas of destruction and the hyper-inflammatory conditions of the lung tissue. The bar chart depicts lung injury scores based on histological analysis of the lung tissues. Data are presented as the mean ± SEM (*n* = 3 mice for the LPS group, 4 mice for the S-protein+LPS group, 3 mice for the S-protein+LPS+TCP-25 group, 3 mice for the S-protein group, and the 3 mice for buffer control). *P* values were determined using a one-way ANOVA with Dunnett’s posttest. **P ≤ 0.01, ****P ≤ 0.0001. (B) Representative scanning electron microscopy images showing changes in the lung tissue 24 h after the intratracheal administration of the combination of the SARS-CoV-2 S protein and LPS. Arrowheads indicate alveolar macrophages.

Dysregulated and excessive inflammatory responses, characterized by the hyperproduction of several pro-inflammatory cytokines and the initiation of different inflammatory signaling cascades, are hallmarks of ARDS and multiorgan failure.^10^ Along with cytokine and chemokine increases, the S-protein–LPS-induced mouse model, with its reductionist approach, also captures features of the complex lung pathology seen in patients with severe COVID-19 in the acute phase. The model could therefore be utilized for analyses of the pathophysiological features of COVID-19 and for the development of new treatments. In a time when new therapeutic approaches for COVID-19 are urgently needed, the simplicity and rapidity of the proposed model is an added advantage.

## Materials and Methods

### Materials

The SARS-CoV-2 S protein was synthesized by ACROBiosystems (USA). The SARS-CoV-2 S protein sequence contains AA Val 16–Pro 1213 (accession no. QHD43416.1 (R683A, R685A)). In brief, the His-tag SARS-CoV-2 S protein (SPN-C52H4) was expressed in human 293 cells (HEK293) and purified. The protein was lyophilized in 50 mM Tris, 150 mM NaCl, pH 7.5. Lyophilized products were reconstituted in endotoxin-free water, aliquoted, and stored at −80 °C according to the manufacturer’s protocol. The purity was >85%. The thrombin-derived C-terminal peptide TCP-25 (GKYGFYTHVFRLKKWIQKVIDQFGE) was synthesized by AmbioPharm (North Augusta, SC). The purity (>95%) was confirmed by mass spectral analysis (MALDI-TOF Voyager, USA). For pulmonary aerosol administration in mice, preparations were made in 50 μL of endotoxin-free water. For coadministration, a 50 μL preparation was made in endotoxin-free water containing 5 μg of S protein and 2 μg of LPS and administered immediately. For pharmacological inhibition with TCP-25, 2 μg of LPS and 20 μg of TCP-25 (molar ratio of TCP-25/LPS 26:1) were mixed in 25 μL of endotoxin-free water and incubated (room temperature, 5 min) followed by the addition of 5 μg of S protein in 25 μL of endotoxin-free water. The dose of S protein was based on previous data derived from both biophysical/biochemical studies and experiments performed on THP-1 cells,^12^ with the latter demonstrating that the S protein was able to increase the pro-inflammatory activity of the ultralow threshold levels of LPS. The dose of 2 μg LPS was based on previously reported *in vivo* data, where it was shown that a doses of 5^25^ and 25 μg^27^ LPS (hence 2.5 and 12.5 times higher than 2 μg, respectively) were required to generate a robust and significant LPS response when injected subcutaneously into mice.

### Pulmonary Aerosol Administration

Female C57BL/6 mice, 8 to 9 weeks old, were used to study the effects of the pulmonary administration of the S protein. Mice were anesthetized using a mixture of 4% isoflurane (Baxter) and oxygen. A specialized pulmonary aerosol delivery device (MicroSprayer Aerosolizer, Penncentury, USA) was used for a precise and air-free intratracheal administration. The mice were suspended on an intubation stand (Kent Scientific, USA) with the help of a loop of suture underneath the upper incisors. Anesthesia was maintained with 2% isoflurane delivered via a nose cone. The aerosolizer was loaded with 50 μL of the solution, with the tip of the device gently inserted down the trachea. The tip of the device was kept close to the first tracheal bifurcation. The aerosolized solution was subsequently administered into the lungs. Thirty seconds after administration, the mice were removed from the intubation stand and returned to the cage. The experiment was terminated 24 h after pulmonary administration, and the mice were sacrificed. BALF was collected (1 mL × 2) and kept on ice. Aliquots of BALF were separated for flow cytometry and cytospin smears, and an aliquot was frozen at −80 °C for the BioPlex cytokine analysis. All animal experiments were performed according to Swedish Animal Welfare Act SFS 1988:534 and were approved by the Animal Ethics Committee of Malmö/Lund, Sweden.

### *In Vivo* Imaging of Inflammation in Mice

Male BALB/c Tg(NF-κβ-RE-luc)-Xen reporter mice (Taconic Biosciences, Albany, NY), 10–12 weeks old, were used to longitudinally monitor inflammation after pulmonary aerosol delivery. Fur from the dorsum was clipped and cleaned. Pulmonary aerosol administration was achieved, as previously mentioned. The IVIS spectrum was used for the bioimaging of NF-κB activation. Fifteen minutes before IVIS imaging, mice were intraperitoneally injected with 100 μL of d-luciferin (PerkinElmer, 150 mg/kg body weight). Bioluminescence from the mouse thoracic area was detected and quantified using Living Image 4.0 software (PerkinElmer). Mice were imaged 6 and 24 h after aerosol administration. Mice were sacrificed after 24 h of imaging, and their lungs, liver, and kidneys were also imaged *ex vivo* using IVIS.

### Flow Cytometry

BD Accuri C6 Plus (BD Biosciences) was used for the flow cytometric analysis of BALF. In brief, cells were washed and stained with Fixable Viability Stain 510 (BD Biosciences) for the differentiation of live and dead cells. Cells were then washed and fixed using Lyse Fix buffer (BD Biosciences). Cells were then washed and divided into two aliquots. A single aliquot was stained with anti-CD11b (BD553312), anti-CD11c (BD558079), and anti-Ly6G (BD551461) antibodies, and the second aliquot was stained with anti-CD11c, anti-MHCII (BD558593), and anti-SiglecF (BD562680) antibodies.

### Cytokine Analysis

BALF collected at 24 h was used for the analysis of lung-specific inflammatory chemokines and cytokines. A Bio-Plex Pro mouse cytokine assay (23-Plex Group I; Bio-Rad, Richmond, CA) was used to assess samples along with a Luminex-xMAP/Bio-Plex 200 system with Bio-Plex Manager 6.2 software (Bio-Rad).

### Histology

Harvested lung tissue was fixed overnight in neutral buffered formalin. After serial dehydration, the tissue was embedded in paraffin blocks, sectioned at 4 μm, and stained with hematoxylin and eosin (H&E). Slides were viewed with bright-field microscopy (Axioplan2, Zeiss, Germany). For histology scoring, an acute lung injury scoring system recommended by the American Thoracic Society was used (Table S1).^28^

### Scanning Electron Microscopy

Lung samples were fixed overnight at 4 °C in ∼10 times the sample volume of “SEM fix” (0.1 M Sorenson’s phosphate buffer pH 7.4, 2% formaldehyde, and 2% glutaraldehyde). After fixation, samples were washed twice in 0.1 M Sorenson’s buffer (pH 7.4) and then dehydrated in a graded series of ethanol (50, 70, 80, and 90% and twice in 100%). Samples were critical-point-dried and mounted on 12.5 mm aluminum stubs and sputtered with 10 nm Au/Pd (80/20) in a Quorum Q150T ES turbo-pumped sputter coater and examined in a JEOL JSM-7800F field-emission gun–scanning electron microscope (FEG-SEM) (JEOL, Japan).

### Statistical Analysis

Differences in the means of more than two groups were compared using a one-way ANOVA with Dunnett’s or Tukey’s post test. Data are presented as the means ± standard error of the mean. Details of statistical analysis are indicated in each figure legend, and GraphPad Prism software v8 was used. *P* values of <0.05 were considered to be statistically significant.

## Supporting Information Available

*Ex vivo* bioimaging of organs in NF-κB reporter mice, *in vitro* inhibition of S protein and LPS synergism by TCP-25, inflammatory mediators in BALF, acute lung injury scoring system, clear-Native (CN)-PAGE, NF-κB activation in human monocytes, cell viability assay, and immunostaining (PDF)
