# Supplementary material for: Experimental Model of Pulmonary Inflammation Induced by SARS-CoV-2 Spike Protein and Endotoxin
Source: ACS Pharmacol Transl Sci. 2022 Jan 25;5(3):141–8. doi: 10.1021/acsptsci.1c00219 (PMC9239546; doi:10.1021/acsptsci.1c00219)
Supplement: Supplementary file 1 — pt1c00219_si_001.pdf [file pt1c00219_si_001.pdf]

Supporting Information

An experimental model of pulmonary inflammation induced by SARS-CoV-2 spike protein and endotoxin

Manoj Puthia [a,*], Lloyd Tanner [b], Ganna Petruk [a], Artur Schmidtchen [a,c]

[a] Division of Dermatology and Venereology, Department of Clinical Sciences, Lund University, SE-22184 Lund, Sweden

[b] Division of Respiratory Medicine and Allergology, Department of Clinical Sciences, Lund University, SE-22184 Lund, Sweden

[c] Copenhagen Wound Healing Center, Bispebjerg Hospital, Department of Biomedical Sciences, University of Copenhagen, DK-2400 Copenhagen, Denmark

[*]Corresponding author. *E-mail address:* manoj.puthia@med.lu.se (M. Puthia).

TABLE OF CONTENTS

Supplementary Figures

Figure S1: *Ex vivo* bioimaging of organs in NF-κB reporter mice…………….**S-3**

Figure S2: *In vitro* inhibition of S protein and LPS synergism by TCP-25……**S-4**

Figure S3. Inflammatory mediators in bronchoalveolar lavage fluid induced by SARS-CoV-2 S protein and endotoxin ……….………………………….……**S-5**

Supplementary Tables

Table S1. Acute lung injury scoring system recommended by the American Thoracic Society…………………………………………………………..…**S-6**

Supplementary methods

Clear-Native (CN)-PAGE…………………………………………………….**S-7**

NF-κB activation in human monocytes ………………………………………...**S-7**

Cell viability assay……………………………………………………………...**S-8**

Immunostaining ………………………………………………………….......**S-8**

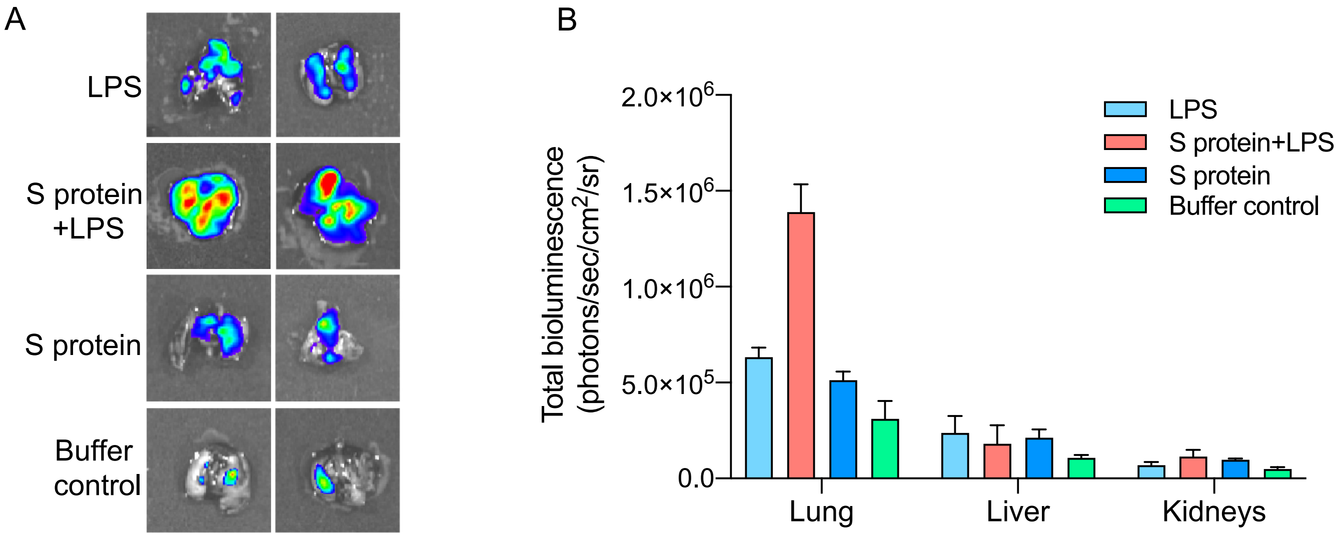

Figure S1. *Ex vivo* bioimaging of organs in NF-κB reporter mice. (A) *Ex vivo* bioimaging of lungs. Representative images show bioluminescence at 24 h after intratracheal administration. LPS alone or in combination with SARS-CoV-2 S protein (S protein) was intratracheally administered in transgenic BALB/c Tg(NF-κB-RE-luc)-Xen reporter mice and bioimaging of NF-κB reporter gene expression was performed using the IVIS. (B) Bar chart shows bioluminescence intensity acquired *ex vivo* from lung, liver and kidneys. Imaging was performed on organs harvested 24 h after intratracheal administration in BALB/c Tg(NF-κB-RE-luc)-Xen reporter mice. Data are presented as the mean ± SEM (n = 4 mice/group).

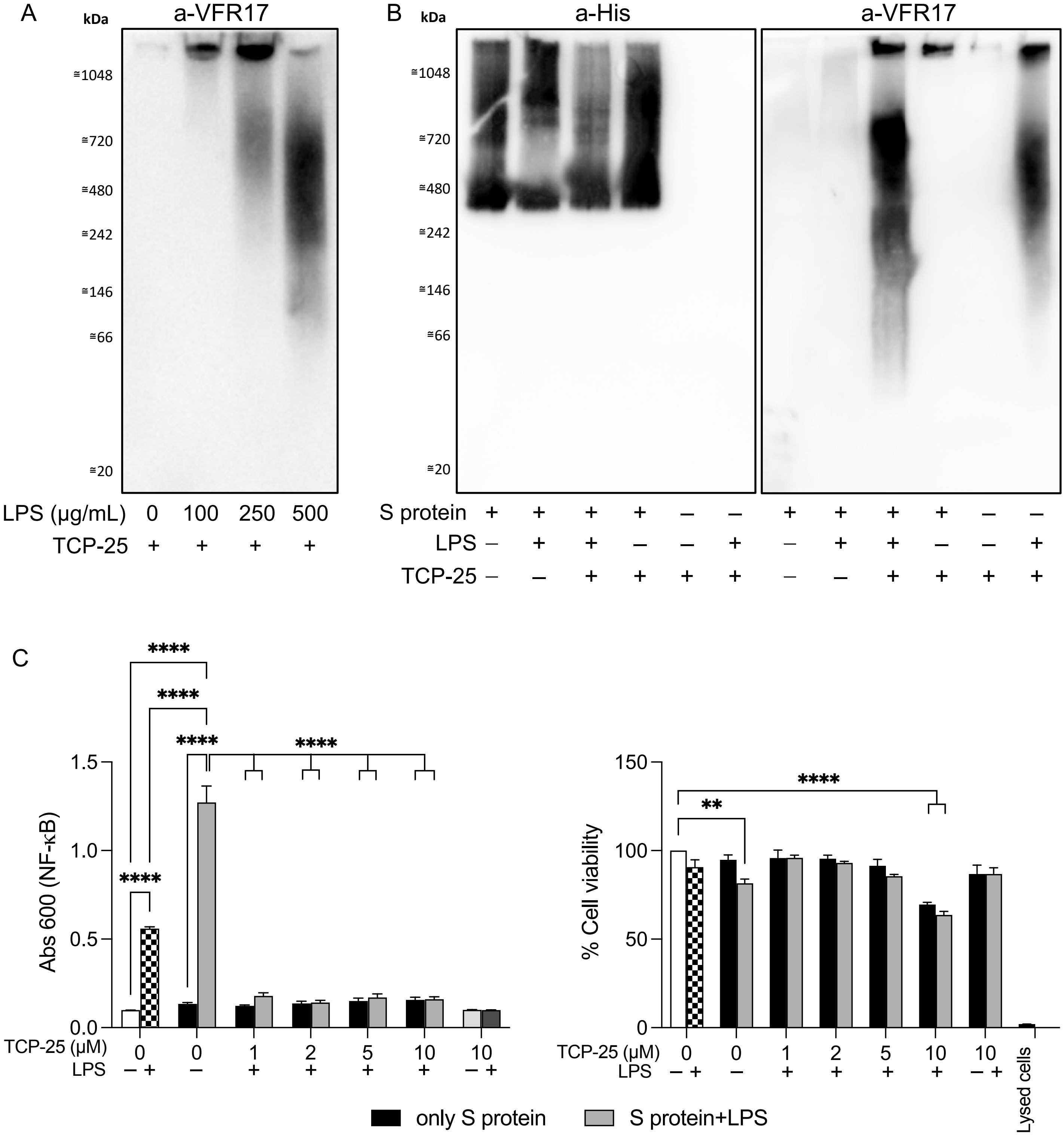

Figure S2: *In vitro* inhibition of S protein and LPS synergism by TCP-25**.** (A) Western blot showing the binding of LPS to TCP-25 with increasing doses (0–0.5 mg/mL) of LPS. One representative image of three independent experiments is shown (*n* = 3). (B) Representative Western blot showing that binding between S protein and LPS is hindered by TCP-25 (*n* = 3). a-His and a-VFR17 indicates that antibodies against the His tag of S-protein and TCP-25 were used, respectively (C) THP-1-XBlue-CD14 cells were treated with increasing doses of TCP-25 (0-10 μM), in the presence of S protein alone (5 nM) or S protein+LPS (5 nM S and 2.5 ng/mL LPS). Histograms on the left show NF-κB activation, while on the right cell viability. TCP-25 blocks S protein+LPS mediated activation. Lysed cells were used as positive control for cell viability assay. The mean ± SEM values of three independent experiments all performed in triplicate are shown. *P* values were determined using a two-way ANOVA with Sidak's multiple comparisons test. $^{**}P < 0.01$, $^{****}P < 0.0001$.

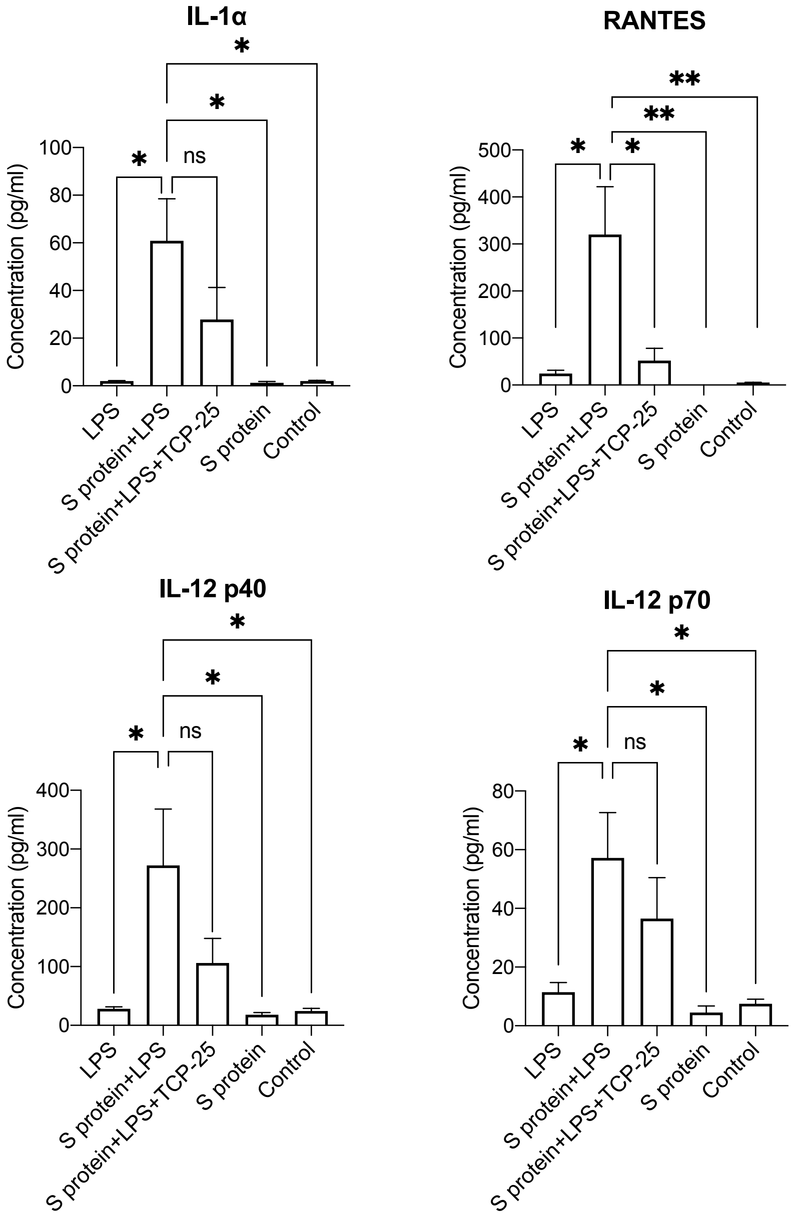

Figure S3. Inflammatory mediators in bronchoalveolar lavage fluid induced by SARS-CoV-2 S protein and endotoxin. (A) Bar charts show cytokines significantly induced by the SARS-CoV-2 S protein and LPS co-administration. Bronchoalveolar lavage fluid was collected at 24 h after the intratracheal administration and analyzed by a mouse a cytokine/chemokine multiplex assay. Data are presented as the mean ± SEM (n = 3 mice for LPS group, 4 mice for S protein+ LPS group, 3 mice for S protein+LPS+TCP-25 group, 3 mice for S protein group and 3 mice for buffer control). P values were determined using a one-way ANOVA with Dunnett's posttest. *$P \leq 0.05$; **$P \leq 0.01$; ns, nonsignificant.

LUNG INJURY SCORING SYSTEM

Parameter		Score per field		
		0	1	2
A.	Neutrophils in the alveolar space	None	1-5	>5
B.	Neutrophils in the interstitial space	None	1-5	>5
C.	Hyaline membrane	None	1	>1
D.	Proteinaceous debris filling the airspace	None	1	>1
E.	Alveolar septal thickening	<2×	2×-4×	>4×

Score = [(20 × A) + (14 × B) + (7 × C) + (7 × D) + (2 × E)]/(number of fields × 100)

Score at least 20 random high-power fields (400× total magnification)

Table S1. Acute lung injury scoring system recommended by the American Thoracic Society.

Clear-Native (CN)-PAGE

TCP-25 (2 μg) was incubated with increasing doses of *E. coli* LPS (0-0.5 mg/mL) at 37 °C for 30 min. Samples were separated under native conditions on CN-PAGE (Native PAGE BisTris Gels System 4–16%, Invitrogen) according to the manufacturer's instructions. Subsequently, the material was transferred to a PVDF membrane using the Trans-Blot Turbo (Bio-Rad, USA). Primary polyclonal rabbit antibodies against the C-terminal prothrombin epitope VFR17 (VFRLKKWIQKVIDQFGE; diluted 1:1000, Innovagen AB, Sweden), followed by swine anti-rabbit HRP-conjugated antibodies (1:1000, Dako, Denmark) were used [27]. The protein was visualized by incubating the membrane with SuperSignal West Pico Chemiluminescent Substrate (Thermo Scientific, Denmark) for 5 min followed by detection using a ChemiDoc XRS Imager (Bio-Rad). In another set of experiments, 2 μg of SARS-CoV-2 S protein was mixed with 0.5 mg/mL of *E. coli* LPS alone or after incubating for 5 min at RT the LPS with 2 μg of TCP-25. Immediately after samples were separated under native conditions as described above and the proteins were transferred on the membrane for Western blotting. The membrane was first incubated with VFR17 antibodies and developed as described above. After stripping, S protein was detected by using primary antibodies against the His-tag (1:2000, Invitrogen) followed by secondary HRP conjugated antibody (1:2000, Dako, Denmark). All experiments were performed three times.

NF-κB activation in human monocytes

THP1-XBlue-CD14 reporter cells were obtained from InvivoGen (San Diego, USA). Cells were cultured in phenol red RPMI, supplemented with 10% (v/v) heat-inactivated FBS and 1% (v/v) Antibiotic-Antimycotic solution. For evaluation of NF-κB activation, 180,000 cells/well were seeded in 96 well plates. Cells were treated with 5 nM SARS-CoV-2 S protein alone, combined with 2.5 ng/mL *E. coli* LPS, or with increasing concentrations of TCP-25 (1-10 μM), or after mixing 2.5 ng/mL *E. coli* LPS with increasing concentrations of TCP-25 (1-10 μM). Cells were then incubated at 37 °C for 20 h. At the end of incubation, the NF-κB activation was analyzed according to the manufacturer's instructions (InvivoGen, San Diego, USA). Data shown are mean values ± SEM obtained from three independent experiments all performed in triplicate.

Cell viability assay

The biocompatibility of the treatments was evaluated by adding 0.5 mg/mL MTT (Thiazolyl Blue Tetrazolium Bromide) to the cells remaining from NF-κB activation assay. After 2 h of incubation at 37 °C, cells were centrifuged, and the medium was removed. Subsequently, the formazan salts were solubilized in DMSO (Duchefa Biochemie, Haarlem) and the absorbance was measured at a wavelength of 550 nm. Cell survival was expressed as percentage of viable cells in the presence of different treatment compared with untreated cells. Lysed cells were used as positive control. Data shown are mean values ± SD obtained from three independent experiments all performed in triplicate.

Immunostaining

Immunostaining of cytospin smears for CD206 was conducted by washing and fixing cells with ice-cold methanol containing 0.5% Triton-X100. Slides were blocked using Dako Protein Block (Agilent, CA, USA) for 1 h at room temperature and then stained with primary rabbit anti-CD206 (1:1000 dilution; Abcam, CAM, UK) antibodies overnight. AlexaFluor 547-conjugated goat anti-rabbit antibody (1:500 dilution; Invitrogen, CA, USA) was used as secondary antibody. Nuclei were counterstained using DAPI-containing fluoroshield (Abcam). Images were visualized using a Nikon Confocal Microscope with fluorescence quantified using ImageJ software.
